# Integrating biology and biography in medicine: The mind and body are not separate

**DOI:** 10.1371/journal.pmen.0000122

**Published:** 2024-09-17

**Authors:** Ralph I. Horwitz, Mark R. Cullen, Allison Hayes Conroy, Ida Sim, Burton H. Singer, Kimberly Badal, Emanuela Offidani

**Affiliations:** 1 Department of Aging and Cardiovascular Discovery, Lewis Katz School of Medicine at Temple University, Philadelphia, Pennsylvania, United States of America; 2 Department of Medicine, Stanford Medical School, Stanford, California, United States of America; 3 Department of Geography and Urban Studies, Temple University, Philadelphia, Pennsylvania, United States of America; 4 Division of General Internal Medicine, and Computational Precision Health, University of California, San Francisco, California, United States of America; 5 Emerging Pathogens Institute, University of Florida, Gainesville, Florida, United States of America; 6 Helen Diller Family Comprehensive Cancer Center, University of California, San Francisco, California, United States of America; 7 Clinical Epidemiology and Evaluative Sciences, Weil Cornell Medical School, New York, New York, United States of America; PLOS: Public Library of Science, UNITED KINGDOM OF GREAT BRITAIN AND NORTHERN IRELAND

It has taken about 400 years, but Medicine is now observing an explicit challenge to Cartesian philosophy, which separates the mind and body For instance, Zeng et. al. recently reported results from a prospective longitudinal study that suggests an association between emotional distress and worse clinical outcomes in patients with advanced non-small-cell lung cancer that is being treated with immune checkpoint inhibitors [1]. The effects were surprisingly large. Although it is possible to interpret these results narrowly for their imlications for the use of immune checkpoint inhibitors, it is also possible to see in these results a deeper appreciation for how lived experience, the patient’s biography, can potentially shape both the risk for disease and the response to therapy.

On the primary endpoint analysis, Zeng et al. report that patients with baseline emotional distress (111 patients) exhibited a significantly shorter median progression-free survival compared with those without distress (116 patients, 7.9 months versus 15.5 months, P  =  0.002). On the secondary endpoint analysis, emotional distress was associated with a lower objective response rate (46.8% versus 62.1%, odds ratio 0.54, P  =  0.022), a reduced 2-year overall survival rate of 46.5% versus 64.9% (hazard ratio for death 1.82, P  =  0.016) and detriments in quality of life. Further strengthening the findings was the evidence that the emotional distress group showed elevated blood cortisol levels, and that the elevations in this stress hormone was associated with adverse survival outcomes [1].

Perhaps a next step is to test a cognitive behavioral intervention in a rigorous trial to see if the negative outcomes associated with emotional distress might be mitigated. If it were, this would be good news for patients with cancers who are receiving immune mediated therapy, and perhaps for cancer patients treated with other therapies.

And yet, perhaps the results Zeng reports shouldn’t be surprising. These results now join an extensive literature linking psychosocial vulnerability to immune medicated diseases. Previous studies have employed a viral challenge model to assess how social, behavioral, and psychological factors made healthy individuals vulnerable to respiratory illness after experimental exposure to cold or influenza virus [2].

Similarly, extensive body of research has demonstrated that vaccine efficacy depends both on the vaccine and the vaccinated. Stress, depression, and loneliness are associated with less robust immune responses to vaccination, and these effects are especially strong among the elderly. Psychological factors have also been demonstrated to alter the prevalence and severity of vaccine side effects [3].

Members of our group previously demonstrated that high adherers to placebo in a randomized controlled trial of beta blockers after heart attack were less likely to die during follow up than poor adherers. Remarkably, the adherence effect in reducing mortality was greater than the effects of the beta blocker itself. These results persisted after adjustment for potential psychological and behavioral confounders [4]. Recently, a network meta analysis of 33 randomized controlled trials showed that patients with COVID-19 treated with standard of care plus placebo had lower odds of all cause mortality than those who received standard of care alone. These studies all point to the powerful influence of mental well-being on beneficial outcomes in randomized trials of dieases possibly mediated through immune mechanisms [5].

The results of all these studies should be understood within the context of research that connects the patient’s lived experience to their biology [6–8]. Diverse biosocial mechanisms allow researchers to hone in on several pathways through which social and physical environments intersect with individual biology to affect health. It is useful to consider one biosocial mechanism that has become central to understanding Biosocial Medicine and perhaps the impact of emotional distress on cancer outcomes—the stress response mediated as allostatic load [9].

Allostatic load, stemming from the concept of allostasis introduced by Sterling and Eyer in 1988 [10], offers a perspective on stress-related research. Allostasis proposes that physiological stability is maintained through variation and predictive regulation of physiological systems in response to stress and orchestrated by the brain. What distinguishes allostatic load from population-level measures of distress is its focus on the individual’s level of stress and their unique response.

Beyond allostatic load, environmental epigenetics has emerged as another major biosocial mechanism for understanding how the external environment broadly affects the internal physiology and risk for disease of individuals. An example of the role of epigenetics comes from elegant studies in flies on the effects of social isolation. Flies were kept in groups or isolation either for an acute interval (1–3 days) or chronic period (5–7 days). Flies kept in groups behaved normally, but flies kept in social isolation had impaired sleep and overate. Remarkably, the investigators were able to identify 214 genes from whole fly heads whose expression was later read, and many of these genes were established causes of both eating behavior and sleep patterns [11].

Biosocial mechanisms also manifest in everyday experiences, shaping physiological responses to emotions like fear and grief. Fear, a powerful emotion, can induce profound cardiac effects acutely, exemplified by Takotsubo cardiomyopathy [12] Similarly, grief—often accompanied by "heartbreak" from the loss of a loved one or divorce—has been linked to increased short-term mortality and long-term disruptions in the regulation of cortisol, blood pressure, and clotting factors [13]. Conversely, positive emotions play a role in shaping biology as well. Finding meaning in life is associated with improved gene expression profiles among individuals scoring high on eudaimonia, a measure encompassing autonomy, mastery, and positive relationships with others [14].

Whether relying on studies to identify molecular and genomic targets for therapy, or randomized trials to separate useful from ineffective medicines, a biological analysis alone is no longer sufficient for the purpose of clinical practice. Each trial needs both a biological and biographical analysis to understand the interaction of biology and biography, and to guide the application of results to clinical practice. What has prevented this integration of biology and biography?

The concept of mind-body dualism, introduced by Descartes in the 17th century, has long constrained and distorted medicine. Descartes proposed that humans consist of two distinct ‘substances’—mind and body—that cannot be unified. According to Descartes, the mind is an immaterial, thinking entity, while the body is material and unthinking [15]. This dualistic view has underpinned the biomedical model dominating medical research and practice. Individuals are often perceived as biological entities, with their constituent elements parsed and understood separately, leading to an entrenched biological reductionism where health is defined as the absence of overt bodily disease.

However, this reductionist perspective neglects the influence of social, psychological, and environmental factors on physical health. Indeed, these distinctions between social, psychological, and environmental are themselves products of dualistic thinking, further separating aspects of human life that are inherently interconnected. By viewing these factors as separate entities, we overlook their interplay and fail to recognize how they emerge as biological processes.

This dichotomous thinking perpetuates a false separation between biosocial mechanisms and biological mechanisms, between biology and biography, and between social determinants and biological determinants. In reality, there is only biology. The body exists within the context of the world, and biography (life experience) is expressed through biology. Contrary to Descartes’ dualism, biology and biography, mind and body, functional and organic, are inherently unified. Recognizing this unity is essential for understanding both the pathogenesis of disease, and the response to treatment.

At the end of the 20^th^ century, researchers began to ask how does the social world “get under the skin,” to affect health. The answer turns the question back at itself. The social world doesn’t simply get into the body, it becomes the body. The social world is already under the skin, because all biology, from womb to tomb, is biographical. All of life’s realities that Descartes deemed immaterial—critical events that impact on physical and mental health like marriage or divorce, birth and death, but also emotional experiences like loneliness, grief, joy, awe and wonder—were considered to be mysterious and external to the material reality of the body. These realities are never external. They are etched in our genome, expressed in our physiology, and contribute to our individual distinctiveness. The implications of this realization are profound.

Zeng’s stunning research findings serve as a clarion call to all investigators to reimagine clinical research. It would be a triumph of patient care if the research reported by Zeng led to improved care for cancer patients with emotional distress. But it would be a scientific tragedy if we fail to seize this opportunity to finally abolish the paradigm that separates mind and body, physical health from mental health, and embrace a new paradigm that studies every disease and every treatment for their potential interactions.
